# Histological evaluation of the influence of magnetic field application in autogenous bone grafts in rats

**DOI:** 10.1186/1746-160X-5-1

**Published:** 2009-01-11

**Authors:** Edela Puricelli, Nardier B Dutra, Deise Ponzoni

**Affiliations:** 1 Oral and Maxillofacial Surgery Unit, Hospital de Clinicas de P.A., School of Dentistry, UFRGS, Porto Alegre, RS, Brazil; 2 School of Dentistry, Federal University of Rio Grande do Sul, Porto Alegre, RS, Brazil

## Abstract

**Background:**

Bone grafts are widely used in oral and maxillofacial reconstruction. The influence of electromagnetic fields and magnets on the endogenous stimulation of target tissues has been investigated. This work aimed to assess the quality of bone healing in surgical cavities filled with autogenous bone grafts, under the influence of a permanent magnetic field produced by *in vivo *buried devices.

**Methods:**

Metal devices consisting of commercially pure martensitic stainless steel washers and titanium screws were employed. Thirty male Wistar rats were divided into 3 experimental and 3 control groups. A surgical bone cavity was produced on the right femur, and a bone graft was collected and placed in each hole. Two metallic washers, magnetized in the experimental group but not in the control group, were attached on the borders of the cavity.

**Results:**

The animals were sacrificed on postoperative days 15, 45 and 60. The histological analysis of control and experimental samples showed adequate integration of the bone grafts, with intense bone neoformation. On days 45 and 60, a continued influence of the magnetic field on the surgical cavity and on the bone graft was observed in samples from the experimental group.

**Conclusion:**

The results showed intense bone neoformation in the experimental group as compared to control animals. The intense extra-cortical bone neoformation observed suggests that the osteoconductor condition of the graft may be more susceptible to stimulation, when submitted to a magnetic field.

## Background

Bone grafts are widely used for oral and maxillofacial reconstructive procedures [1]. The influence of electric fields, electromagnetic fields and magnets on the stimulation of endogenous mechanisms in tissues is under research [2-5], in situations such as the repair of bone fractures with pseudoarthrosis, integration of bone grafts, osteoporosis and osteonecrosis [6-8]. Electromagnetic fields may influence different cell functions [9-11].

Electromagnetic fields may be applied with specifically designed devices, composed of spirals connected to a pulse generator. When the generator is turned on, electric current circulates and a magnetic field is established between the spirals. This type of electromagnetic field has been used for the stimulation of connective tissue repair [7], and has shown positive results in the treatment of fractures in humans [6,8,12].

Bruce and colleagues [2] investigated the effect of magnetic fields of 220 to 260 Gauss (G), produced by externally placed samarium cobalt magnets, on fracture healing in rabbits. Bone healing was assessed microscopically and mechanically, four weeks after the surgery. The bone exposed to magnetic fields were more resistant to breaking than control bone, but no significant difference was observed between magnetized and control groups.

Other studies, however, have shown controversial results on the influence of magnetic fields on tissue repair. Linder-Aronson and Lindskog [13], for instance, reported bone resorption in the tibia of rats near to implanted samarium cobalt magnets.

Puricelli and colleagues [14] evaluated histologically the influence of static magnetic fields produced by stainless steel washers buried in the bone, adjacent to a surgically created cavity in rats. In the control group, washers were not magnetized. The animals were sacrificed 15, 30, 45 and 60 days later, and samples were collected and histologically analyzed. Samples from the experimental group showed extensive trabecular formation beginning in the endosteum (day 15), formation of compact bone with a tendency to centripetal growth (day 30), and increased osteoclastic activity and bone remodelling (day 45). On day 60, experimental samples showed marked external configuration of the cortical bone surrounding the magnetic washers, with bone formation surpassing the cortical level. These results showed that magnetic fields, in this experimental model, resulted in increased efficiency of the experimental bone healing process.

Few studies have assessed the influence of magnetic fields on bone healing after autogenous bone grafting. Improved integration of bone grafts by the stimulation of the receptor site and the graft with the use of magnetic fields may represent an important clinical advancement, particularly in Oral and Maxillofacial Surgery, Osteointegrade Implants and Orthopedics.

## Methods

This randomized experimental study, aiming to evaluate the influence of permanent magnetic fields buried *in vivo *on autogenous bone grafts, used methods previously reported by Puricelli et al [14] and Ulbrich [15]. Thirty male Wistar rats (*Rattus norvegicus albinus*), 5-month old and weighing in average 400 g, were used. They were divided into 3 experimental and 3 control groups, which were analyzed on days 15, 45 and 60 after beginning of the experiment.

The metal devices consisted of commercially pure martensitic stainless steel washers and titanium screws. The screws measured 1.0 mm in diameter, 0.5 mm in thread pitch and 2.0 mm in length. The pre-made magnetized washers were 3.0 mm in outer diameter, 1.5 mm in core diameter and 0.5 mm thick. They were held over a 60 mm × 12 mm × 5 mm magnet during the sterilization process and surgery. Magnetic champs calculations were performed at the Electromagnetism Laboratory, Physics Institute from Universidade Federal do Rio Grande do Sul.

**Figure 1:**
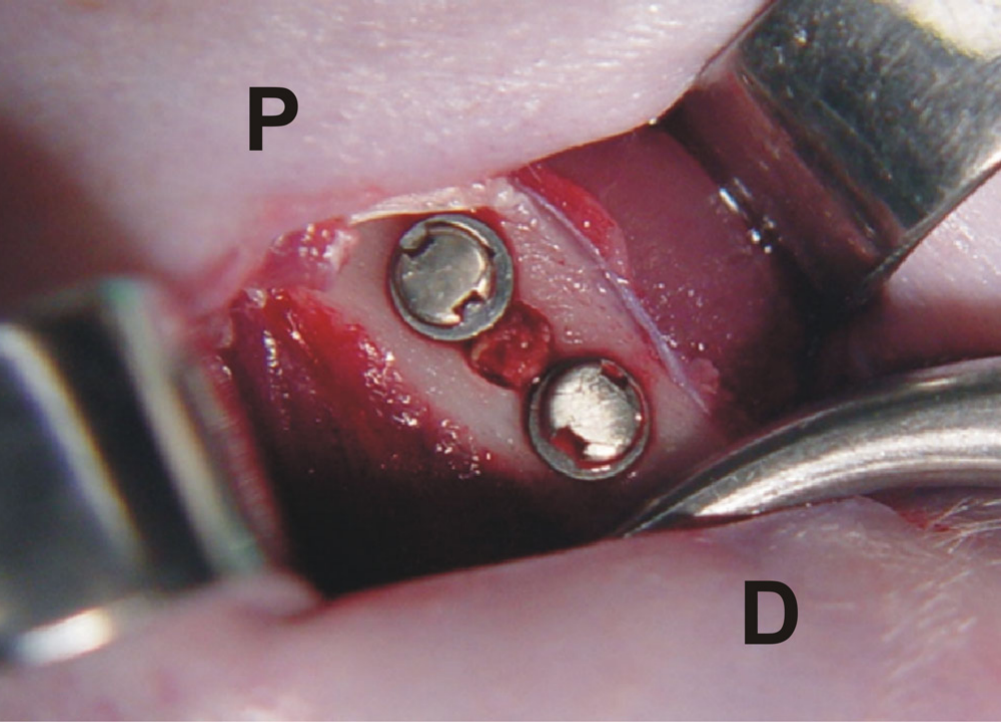
**Screws and washers outlining the borders of the surgical bone cavity, in which the bone graft is placed**. The washers are 1.3 mm apart, limiting the area where the magnetic field operates. P and D, proximal and distal regions of the right femur, respectively.

The animals were anesthetized by intramuscular injection of ketamine and xylazine at 0.1 ml/kg and 1.0 ml/kg body weight, respectively, and local infiltration of 3% prilocaine with felypressin. After reaching the medial portion of the right femur diaphisis, a surgical bone cavity was produced with a trephine (PROMM^®^, Comércio de Implantes Cirúrgicos Ltda., Porto Alegre, RS, Brazil) measuring 2.0 mm diameter active region, with low rotation and constant irrigation. Two holes were drilled with a drill guide (PROMM^®^), at 1.0 mm from the ostectomized border, one of them proximal and the other one distal to the surgical bone cavity. The corticospongeous bone graft was delicately removed from the trephine with the aid of a probe, and placed vertically into each of the 2.0-mm holes (Figure 1). The washers were attached to the bone structure with titanium screws. A magnetic field was established in animals of the test groups by placing up the north and south poles of the distal and proximal washers. In control animals, the surgery was performed with non-magnetized instruments, washers and screws.

The placement and stability of implants and bone grafts were confirmed by radiographic control at the end of the experiments. After sacrifice of the animals, femurs were longitudinally sectioned, which allowed simultaneous examination of the surgical cavity between the screw holes. Samples were collected and prepared in hematoxylin and eosin stain (HE) for histological analysis.

## Results

**Figure 2:**
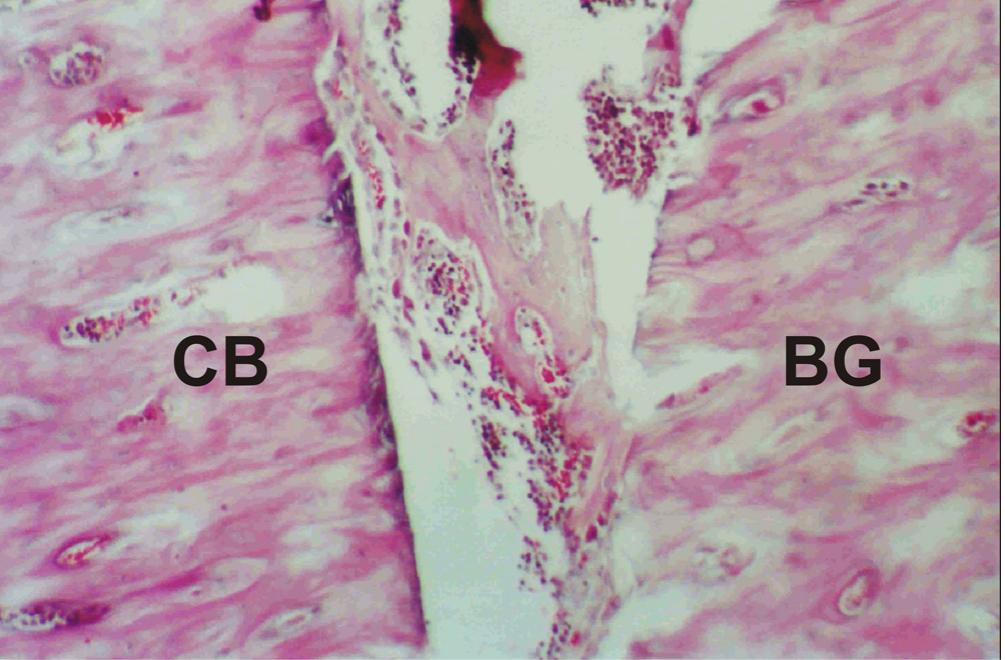
**Control group, day 15**. Proliferating bone structure connecting the cortical bone (CB) border to the bone graft (BG). (HE, 400×).

**Figure 3:**
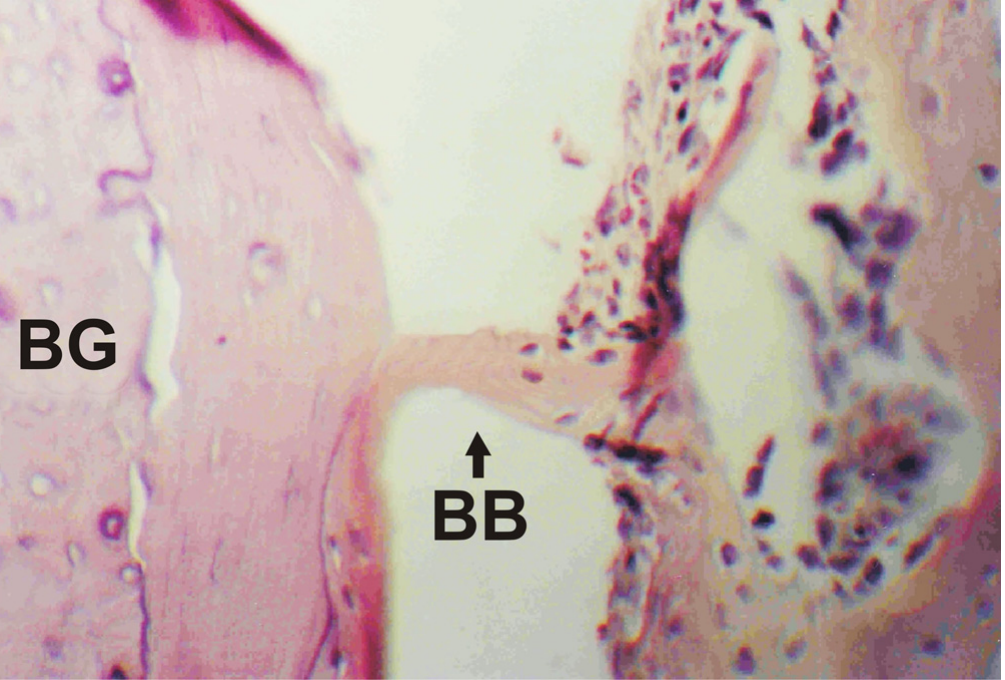
**Control group, day 15. Bone bridge (BB) linking the bone graft (BG) to the healing region**. A large number of osteoblasts may be seen migrating from the cortical bone to the graft. A lamellar bone outline, continuous to the neoformed bone structure in the graft, may also be observed. (HE, 400×).

On day 15, histological analysis of control samples showed bone neoformation, beginning on the endosteum and surrounding the surgical cavity, within which the grafts could be seen in vertical position. Active areas of angiogenesis, indicative of bone health, were observed. At higher magnification, a bone structure with apparent proliferative activity was observed linking the cortical border to the graft (Figure 2). Samples from the experimental group showed good stability of the bone grafts, which could also be observed vertically placed in the surgical cavity. Areas of neoformation of spongeous or trabecular bone were less frequent, with progressive replacement by hematopoietic marrow. Vascular structures were present in the interface between the residual bone and the graft, and mature medullary tissue was observed. An osseous bridge was seen connecting the graft to the bone neoformation area (Figure 3). Other histological aspects included large numbers of osteoblasts migrating from the fixed structure to the graft and the formation of lamellar outline structures in the graft.

**Figure 4:**
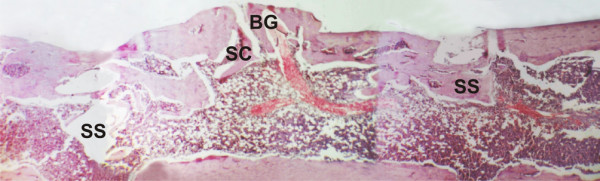
**Control group, day 45**. Horizontal composition of pictures showing the sequence of the surgical cavity (SC) and screw space (SS). The screw holes and the surgical cavity between them may be observed. Bifurcating blood vessels invade the healing region and the bone graft (BG). Leveling of the cortical continuity, with slight extrusion of the grafted area, is observed. (HE, 40×).

**Figure 5:**
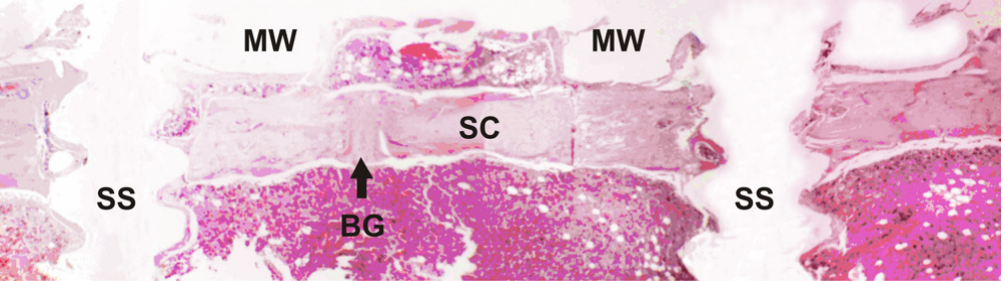
**Experimental group, day 45**. Horizontal composition of pictures showing the sequence of the surgical cavity (SC) and screw space (SS). The autogenous bone graft (BG) contributes to the cortical closure of the surgical wound. A marked vertical positioning of the bone graft is observed, reflecting its original position. Exophytic growth of the bone structure superimposed on the surgical wound, outlining the area between the magnetized washers (MW). (HE, 40).

The histological analysis of control samples on day 45 showed bone neoformation, beginning in the cortical border of the surgical cavity and involving the graft. Mature osseous tissue was seen in this region of the cavity. Large blood vessels were observed in the medullary canal, in the direction of the cortical bone and graft areas. Areas of spongeous trabecular bone were progressively replaced by mature hematopoietic marrow, as shown by the presence of adipocytes (Figure 4). In samples from experimental animals, residual graft tissue could be seen in the region originally engrafted, integrated to the cortical structure in vertical position. Intraosseous spaces, with cellular and vascular activity, were observed in the graft and in the residual cortical. Bone neoformation was clearly seen, beginning in the periosteum and having a centrifugal direction, parallely superimposed on the cortical cicatrization which was kept in its original level. The neoformed bone limited with the buried magnetized washers, in both borders of the surgical cavity. Active hematopoietic tissue was observed, with intense vascular proliferation in the new medullary space surrounded by the neoformed bone. This structure was similar to the medullary structure of the bone conduct (Figure 5). In some specimens, images possibly representing marginal sectioning of the samples were observed, with large amounts of cortical bone and little medullary tissue. The growing bone tissue, centrifugally directed, presented a well delimited cortical structure which marked clearly the borders of the magnetized area. The same orientation was observed for the vascular structures, which originated in the femur bone and were directed to the neoformed bone region, with the periosteum tightly surrounding the whole area. A thin capsule of fibrous connective tissue could also be observed near the washers.

**Figure 6:**
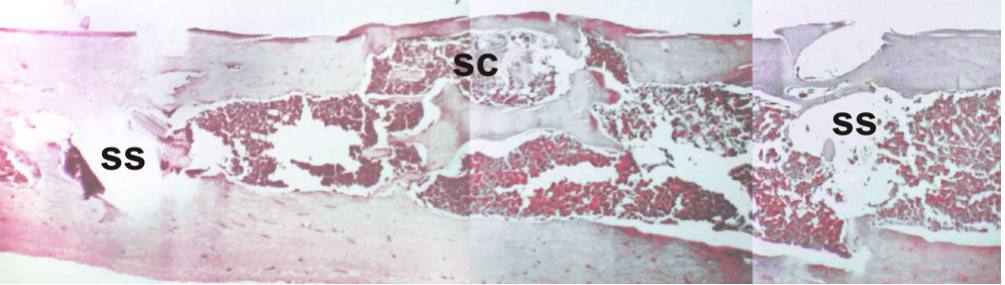
**Control group, day 60**. Horizontal composition of pictures showing the sequence of the surgical cavity (SC) and screw space (SS). Continuity of the cortical structure indicates the healed central area, placed between the screw spaces. Osteoclastic activity is observed, beginning in the marrow and reorganizing the medullary canal. (HE, 40×).

On day 60, histological analysis of control samples showed that, in the graft area, the surgical cavity was filled with slightly convex cortical, without clear visibility of the washers borders. In some of the specimens, the thin cortical structure with massive presence of marrow was apparent. No structures representing the autogenous bone graft could be seen (Figure 6). In samples from experimental animals, the new cortical showed a tendency to remodelling, in a process beginning in the neoformed region. Other features included invasion of the primary cortical compact structure by bone marrow, and the presence of many Howship's lacunas, characterizing progressive resorption. No structures representing the autogenous bone graft were observed.

## Discussion

The present study followed the research line established by Puricelli [14]. As in many other experimental studies, the rat was used as a model, due to its advantages in terms of ease of acquisition, maintenance and surgical manipulation [16-19]. The washers were maintained in place, bordering the site of the bone graft, by commercially pure titanium screws used in the experiments. The biocompatibility of titanium was confirmed in this study, as already observed by Puricelli et al [14] and Ulbrich [15].

Most of the studies in this field investigate the influence of electromagnetic fields on bone healing pseudoarthrosis or delayed healing [6,8,12]. In most of them, the magnetic fields are created by specifically designed devices external to the animal [2,13,20,21]. This system presents some disadvantages such as the need of daily application of an electromagnetic field, long duration of the treatment and the need to connect to a source of electricity during application of the electromagnetic field [12]. The pioneering work by Puricelli et al. and Ulbrich in 2003 [14,15] introduced the concept of static magnetic fields arranged inside the body, which resulted in increased efficiency of the experimental bone healing process.

The experimental design and selection of methods to investigate the influence of magnetic stimulation on tissue repair are hampered by the scarcity of reports in the literature and lack of consensus on the intensity of the magnetic fields to be tested. The intensity of magnetic fields used in different studies ranges from 2 × 10^-4 ^T (Tesla) to 8 T. This large variation is due, among other factors, to difficulties in the production and adjustment of the devices to create magnetic fields with intensities within the therapeutic range. Studies have also shown great variability in the duration of applications and treatments as a whole, with reports of daily applications ranging from one to eight hours, and treatments ranging from two days to eight weeks [6-8,11,21].

The present work included a pilot study based on the method described by Puricelli [14] (data not shown). Measurements of the magnetic fields in ten femurs showed that mean initial intensities on days 0, 15 and 60 were 51.52 × 10^-4 ^T, 43.83 × 10^-4 ^T and 25.36 × 10^-4 ^T, respectively. During the experiment, the field was active but variations on the intensity were observed. These individual variations may represent methodological artifacts, due to the methods used for measurement, or they may be explained by differences in the composition of the metallic washers. The maintenance of a permanent magnetic field, buried in the tissue, opens the possibility of investigating the effect of continuous activity of magnets on the osseous tissue.

Our results showed that, on day 15, grafts were viable in control and experimental animals, but the interface of neoformed bone with the graft was more evident in the experimental group. Similar results were obtained by Puricelli et al [14] and Ulbrich [15], with intense trabecular formation in control animals on day 15, when compared to the control group. On day 45, samples from experimental animals presented only remnants of the grafts, and intense bone neoformation beginning on the periosteum parallely superimposed to the scar cortical, evidencing the osseous borders of the magnetized washers. These results show that bone activity was higher in experimental rats, when compared with control animals in the same period. On day 60, the new cortical on the magnetically stimulated graft area showed a tendency to remodelling, beginning on the neoformed region. The cortical showed a slightly convex configuration in control samples. This study thus suggests that the magnetic field, associated to the osteoconductor ability of the bone graft, induces areas of bone neoformation apparently larger than those previously observed by Puricelli and colleagues [14].

## Conclusion

Taken as a whole, our results showed that:

1. The magnetized stainless steel washers used in this work influenced positively the integration of the bone grafts.

2. The histological analysis of the region of bone graft on postoperative days 15, 45 and 60 showed that the permanent magnetic field stimulates by itself bone neoformation.

3. The intense extra-cortical bone neoformation observed in the experimental group suggests that the osteoconductor factor of the graft may be more susceptible to stimulation.

## Competing interests

The authors declare that they have no competing interests.

## Authors' contributions

EP conceived of the study, participated in its design and coordination. NBD participated in the design of the study and the experimental steps. DP carried out the experiments and analyses. All authors helped to draft the manuscript and approved its final form.
